# Chicago Medical Society

**Published:** 1878-03

**Authors:** 


### CHICAGO MEDICAL SOCIETY.

Regular meeting, Monday, January 21, 1878. President, E. Ingalls, in the chair; 23 members present.

*Fibro-sarcoma of lower lid; Extirpation and Plastic Operation.*—Dr. F. C. Hotz exhibited a patient aged 54 years, who had come under his care one week ago. He had under his left lower eyelid a round tumor of the size of a quarter dollar: its upper circumference reaching within one-eighth of an inch to the free edge of the lid. The tumor had been growing from a small wart to its present size during the past two months. It was excised, and the triangular defect was covered by the sliding of a flap of integument borrowed from the malar-temporal region. All wounds were closed by fine silk sutures and healed by first union. The morbid growth, which was also exhibited, was limited to the cutaneous tissues, showing a marked hypertrophy of the papillæ, and under the microscope the structure of a fibro-sarcoma.

*Heart Disease and Ebolism.*—Dr. M. H. Thompson read an elaborate history of the following case: The patient, aged 41, was admitted to the Woman's Hospital, and on November 12th, delivered of a seven month's child. It was her third pregnancy. For six years she had been subject to epileptiform convulsions. With the exception of a slight chill on the fourth day, the patient was doing well until the fourteenth day, when she had a slight fainting fit, supposed to be one of her epileptic attacks; she was found on the floor with the whole right side paralyzed, and no pulsation in right arm and foot, nor in the right carotid artery. The patient became comotose, and died on the eighteenth day after confinement.

The autopsy showed a narrowing of the left auriculo-ventricular opening, so as to admit only the point of the finger; but there was no calcification. A large white fibrous clot in the left ventricle adhered to the wall. A clot was also found in the right common carotid, the right vertebral and left middle cerebral arteries.

(The report did not state whether the right sub-clavian and right iliac or femoral arteries had been examined, in order to detect the causes of the interruption of circulation in right arm and leg.—R.)

*Death from a Mixture of Ether and Chloroform.*—Dr. H. VanBuren reported the death of a lady, aged 32, and a confirmed opium eater, from the inhalation of a mixture of two parts of ether and one part of chloroform. The patient, as the doctor learned *post festum*, had taken a large dose of morphia a few hours previous to the administration of the the anæsthetic. No autopsy.

Some general remarks were made by a few members concerning the relative safety of ether and chloroform, and then the discussion turned chiefly upon the use of chloroform in labor cases.

---

Regular meeting, Monday, February 4th, 1878. President, Dr. E. Ingals, in the chair; 15 members present.

*Syphilitic Lesions of the Nerve-Centres.*—Dr. S. D. Jacobson read a paper which, in a very able manner, presented the present state of knowledge of the subject.

Owing to their curability, the luetic affections of the cerebrum do not often give an opportunity to examine into their anatomical basis. But we may judge of the cases cured from analogy with those that end fatally. In those cases we find lesions of various kinds and in different localities; changes in the bones, the membranes, the nerve substance, and in almost any locality; often they are multiple, sometimes result after pathological processes to be found also in non-syphilitic patients, as hemorrhages, inflammations, anomalies of nutrition; partly are they of a more specific character, especially gummata.

Heubner was the first to call attention to the changes in the cerebral arteries in syphilis. They undergo changes in the thickness of their walls, in their capacity and permeability—changes which, of course, were often overlooked at the *post mortem.* The alteration of circulation produced by those morbid arteries explains the symptoms, and especially their multiformity and variety. In this manner we may understand why severe symptoms sometimes appear suddenly, and that, owing to the removal of obstacles to the circulation, the worst incidents admit of a speedy amelioration.

For the diagnosis of the syphilitic nature of cerebral disorders, it is very important to establish the infection and the previous or present existence of other syphilitic symptoms. And yet, we must bear in mind that a syphilitic patient may become afflicted with cerebral disorders which have no relation to the constitutional disease. It would, therefore, be very desirable to establish the diagnosis from the nervous systems, or at least the prevalence of certain symptoms over others. But, unfortunately, there is not one single symptom which is, *per se*, pathognomonic of the syphilitic nature of a nerve lesion. Every anomaly of function which depends upon cerebral or spinal trouble may be found in cerebral lues, and every single symptom which is found in the latter, may also be found without syphilis. Lues may produce disorders of sensibility and motility of every description, from the slightest abnormal sensation to the most intense pain; from a scarcely perceptible numbness to a complete anæsthesia of every organ of sense; every variety of involuntary movement to epileptiform convulsions; and again, debility from the slightest degree up to complete paralysis of every cerebral or spinal nerve; insomnia as well as somnolence unto the profoundest coma. Disturbance in the psychical functions, abnormal excitement unto raving mania, as well as dullness of various degrees, of the memory, of the mental power, of the judgment, of the will power to complete dementia. In all of those cerebral and spinal symptoms there is nothing specific to syphilis, either in mode or in degree.

We may suspect a syphilitic basis of nervous symptoms if they appear suddenly without any apparent direct cause; if they exhibit a peculiar incompleteness in their development, as, for instance, epileptiform convulsions without loss of consciousness; and especially if symptoms appear in a combination which cannot be produced by one single diseased focus (for instance: ptosis in one eye, and paralysis of external rectus of the other eye; paralysis of the motor oculi and of the facial nerve of the other side; convulsions with paralysis). Very suspicious, also, are the rapid changes of character in the symptoms, such as are found only in one other disease; i. e., hysteria.

In many cases we find, for some time, insignificant symptoms which are overlooked, and disappear from time to time; such are fainting spells, sudden but momentary aphasia, a sudden neuralgia. On account of their momentary duration, the patient himself generally pays but little attention to them. This stage we consider a premonitory stadium. More severe troubles now set in, generally in a sudden apoplectiform manner; such are fainting, sudden aphasia, epileptiform or apoplectiform attack, sudden partial paralysis, blindness; or they may increase in number rapidly, as acute mania or melancholia, stupor, intense headache or other neurlgia, choreic and cataleptic fits, paraplegia.

Quite frequently we observe symptoms which in non-syphilitic affections of the nerve-centres indicate impending dissolution, (as stupor, aphasia, hemiplegia) in specific cases disappear spontaneously, in spite of the poor general condition of the patient; also, that parts which first appear paralysed regain their mobility, while in the opposite half the body a ptosis, a paralysis of the arm or the like may appear, so that the gravest symptoms alternate, which never could take place if dependent upon an extravasion of blood, or other destruction of the corresponding cerebral locality.

With the exception of the most acute and the too inveterate cases, cerebral syphilis stands a fair chance for successful treatment; spinal lues much less so. By an energetic treatment with iodide potassium or mercury, assisted by hot sulphur baths, we generally have it in our power to alleviate the worst symptoms, and very often to cure the case. In fact, it may justly be said of a patient with severe cerebral symptoms, that his chances for a cure are infinitely greater if he be syphilitic, than if he is not. Many cases that do not seem to improve much in their homes, improve rapidly if sent to hot springs, in combination with specific treatment. In spinal syphilis our success is much rarer and less complete.

In the discussion of the essay Dr. Logan developed his view of the nature of constitutional syphilis in the following words: That there exists a specific virus in connection with each of the contagious diseases, endowed with the property of reproducing itself, there can be no doubt. But his conception of it was not that of an entity which diffuses itself throughout the system. The virus represents in its molecular construction a certain type of morbid action and has the power of inducing and continuing this action in an economy which has not already undergone it. When this virus is brought into contact with a healthy person, it at once sets up an action which ultimately results in assimilating the whole molecular structure of the subject to that of its own. A man, therefore, who has had a non-recurrent contagious disease, atomically and molecularly considered, is a different individual from the one he was before the attack.

Entertaining these views of the whole class of contagious diseases he had no faith in efforts to eliminate from the system the poison of syphilis; and our specific remedies for that purpose were only operative against the morbid movement involved in the diseased action which we called syphilis. The disease is constitutional from the moment the specific virus begins to induce molecular change in the contagious structures; and the primary sore is but the focal expression of the general disease. Why the former should appear at the point of application of the virus is applicable upon the same basis which underlies the production of the vaccine pustule at the point of insertion. The theory of a mere local irritation was barred by the inability to produce a second specific sore upon the same person by inoculation with the specific virus of the first.

---

Regular meeting, Monday, February 18th, 1878. President Dr. E. Ingals in the chair. Twenty-nine members present.

*Shall the forceps be applied with reference to the pelvis or to the position of the child's head? Primary or secondary operation for ruptured perinæum?* A discussion on these two questions followed the report of a case of protracted labor, related by Dr. S. Baker. The woman had been in pains over twenty-four hours; the os uteri was dilated; pelvis narrow and child's head very large. Labor pains did not succeed in expelling the head. Chloral in 40 gr. doses had no effect; ergot failed. The doctor then tried to apply forceps with regard to the head, but failed; while, when afterwards, the instrument was applied with reference to the pelvic cavity, the blades could easily be locked; a complete rupture of the perinæum resulted, but the wound was immediately closed by deep silver sutures.

Dr. Van Buren expressed his preference for applying the forceps with reference to position of head, and for the immediate application of sutures to a ruptured perinæum.

Dr. T. D. Fitch thought the weight of authority was in favor of applying the forceps with reference to the pelvis only. The position of the head could be improved in many cases by external and internal manipulations. As to perinæal lacerations, he considered the secondary operation the more judicious procedure, because the new shock by the immediate operation upon the already exhausted nervous system of a puerpera might—and in the only case in which he did the primary operation, the Doctor thought it did—have a fatal effect; also the necessity of a continued constipation would act unfavorably upon puerperal patients. While on the other hand, he did not find that the exposure of a large raw surface to the puerperal secretions increased the danger of puerperal fever.

Dr. Marguerat followed the very simple rule: Apply the forceps the best way you can, and if they lock you may be sure they are properly applied.

Dr. Maynard had found ergot not so infallible in its action as most physicians were disposed to believe. In one case, the contractions of the womb ceased after the expulsion of the child, and could not be induced by external and internal manipulation, ice in utero, ergot and electricity; but they were speedily and energetically produced by one ounce of whisky. When the effect of this dose was spent, the uterus began again to relax, and nothing short of a repetition of the dose of whisky would stimulate it.

Dr. E. F. Ingals mentioned a case of *post partem* hemorrhage, due to atony of the uterus, which was speedily arrested by injections of hot water.

Before adjourning, the president appointed a committee of three to confer with a similar committee of the Society of Physicians and Surgeons, in reference to uniting the two medical societies, and suggesting such changes in the plan of organization as they might deem practicable. —*Chicago Medical Journal and Examiner.*

